# Statistical Learning and Social Competency: The Mediating Role of Language

**DOI:** 10.1038/s41598-020-61047-6

**Published:** 2020-03-04

**Authors:** Kaitlyn M. A. Parks, Laura A. Griffith, Nicolette B. Armstrong, Ryan A. Stevenson

**Affiliations:** 1 Western University, Department of Psychology, London, ON Canada; 2 Western University, Brain and Mind Institute, London, ON Canada; 3 Western University, Program in Neuroscience, London, ON Canada; 4 Western University, Department of Psychiatry, London, ON Canada; 5 York University, Centre for Vision Research, Toronto, ON Canada

**Keywords:** Human behaviour, Signs and symptoms

## Abstract

The current study sought to examine the contribution of auditory and visual statistical learning on language and social competency abilities as well as whether decreased statistical learning abilities are related to increased autistic traits. To answer these questions, participants’ (*N* = 95) auditory and visual statistical learning abilities, language, social competency, and level of autistic traits were assessed. Although the relationships observed were relatively small in magnitude, our results demonstrated that visual statistical learning related to language and social competency abilities and that auditory learning was more related to autism symptomatology than visual statistical learning. Furthermore, the relationship between visual statistical learning and social competency was mediated by language comprehension abilities, suggesting that impairments in statistical learning may cascade into impairments in language and social abilities.

## Introduction

Over time, we learn which pieces of information belong together or follow one another more frequently and are then able to identify these relationships by uncovering the probabilistic information occurring within them^1^. This process, known as statistical learning, functions implicitly^2,3^ and allows individuals to track patterns and probabilities within the environment, and to predict what pieces of information will come next^4–6^. Such probabilistic information permeates much of the social world, from recognizing the nuances of body language, to learning associations between words and their appropriate meanings, to social turn taking in a conversation. The ability to discover these underlying regularities helps learners make sense of their world by finding structure in a rapidly changing, continuous environment.

Statistical learning is important for understanding patterns in social interactions^7–9^, including how to respond to social events and/or how to adjust one’s behaviour accordingly. It also plays an important role in facilitating language skills including syntax^10^, semantics^11^, word segmentation^12^, and early literacy skills^13^. For example, in English, appropriate syntactic structure can be signaled by highly probable cues such as the word *the* being followed by a noun. Another example is spoken sentences that have highly predictable and non-predictable endings such as the predictable ending *prize* in *her entry should win first prize* and the non-predictable ending *beach* in *the arm is riding on the beach*^14^. Knowledge of such probabilities in spoken language can help a listener identify or predict the next word in a sentence. This probabilistic structure can also help learners recognize syntactic violations as well as better understand word meanings and appropriate sentence structure.

Perhaps the most studied area of statistical learning is in spoken language. Certain syllable pairs occur more often in English (and in other languages as well) but, unlike written words, it is not always clear when one-word ends, and another begins in spoken language. A classic example of this is the phrase *pretty baby*. In natural speech, there is no clear pause between these words. However, over time, we learn to perceptually separate these words, a process known as stream or word segmentation^15^. One reliable way to segment words is to use a clustering mechanism based on the conditional probabilities between syllables^16^, or tracking the co-occurrence frequency between syllables. In other words, learners can segment words by uncovering and predicting the statistical patterns that occur between syllables. Statistical regularities are present in words within natural languages, making certain syllables more predictive than others, helping learners determine when one-word ends, and another begins. In turn, this probabilistic information about appropriate language structure can help language learners understand words, word meanings, and sentence structure better^17^. Syllables within the same word typically have higher conditional probabilities; they tend to co-occur more often than syllables that occur between words. This high and low probability of co-occurrence can be formally described as the probability of *Y* given *X*^18^:

$$p(Y|X)=\frac{p(X{\cap }^{}Y)}{p(X)}$$

Although this process may seem computationally demanding, research has shown that language learners can use transitional probabilities to reliably segment words from fluent speech^19–21^. Previous research has demonstrated that even infants can segment words from fluent speech using only transitional probabilities^19^ and this finding has been widely replicated in more recent studies using both artificial^20,22–26^ and natural languages^27,28^.

Although the majority of statistical learning studies have been conducted in the auditory domain, statistical learning is not limited to this modality. The need to segment information into meaningful patterns extends to the visual domain as well^29^. One of the first studies to examine whether statistical learning can operate in the visual domain found that infants as young as 2-months old were able to extract the statistical patterns in a sequence of visually presented shapes^30^. In this study, infants were presented with a series of colourful shapes that followed a predictable pattern. Pairs of shapes that occurred together more often had higher transitional probabilities than those that occurred between shape pairs. Learning was measured through looking times where, as expected, infants showed longer looking times to the novel shape pairs compared to the familiar pairs. These findings, along with other research showing that infants and adults are able to learn statistical patterns presented in tone sequences^31,32^ indicate that statistical learning is occurring across modalities whereby learners are able to use transitional probabilities to segment sequences across multiple sensory modalities.

Following these findings that statistical learning occurs early in life, much subsequent attention has been given to the role that it plays in early language learning. A longitudinal study examining the relationship between early speech segmentation and later vocabulary outcomes found that at age two, children with poor vocabulary skills were less successful at segmenting the speech stream during infancy^33^. Early segmentation ability was significantly related to children’s semantic and syntax abilities in the preschool years^33^. Further, visual statistical learning has been implicated for its role in language proficiency skills including syntax^34,35^, early literacy skills^13^, and receptive language ability^36^ while auditory statistical learning is involved in grammar^37^, language processing^38^, and vocabulary size^39^. In particular, research investigating the link between visual statistical learning and language has found that children who show stronger visual statistical learning abilities are better able to detect and adhere to rule changes in syntactic structure^34^. Therefore, it appears that children who are better at identifying patterns and regularities are also better able to apply these patterns to other areas of language, in this case, sentence structure.

Though not as thoroughly studied, emerging research has suggested that statistical learning may play a role in some aspects of reciprocal social interactions, including predicting behavioural patterns^7^. Such research has examined whether adults could uncover statistical regularities to predict dynamic, intentional activity in humans. Participants were exposed to a continuous silent video of human actors performing predictable tasks. It was found that adults could successfully segment human action using statistical regularities, highlighting the important role that statistical learning plays in identifying and understanding the actions of others^7^. A stronger ability to analyze and predict the current and future actions of others have been hypothesized to lead to more effective social interactions. It may also be that statistical learning helps learners determine how they should respond or react to another individual or during a social event. For instance, it has been suggested that those better at detecting behavioural patterns or relationships are also better able to correlate these relationships with the goals and/or intentions of another individual, helping them to respond accordingly. Indeed, past research has demonstrated that adults can successfully uncover statistical regularities that provide information about intentionality in human action^7^ and that auditory and visual processing is related to non-verbal communication in non-ASD and ASD children^40^. Additionally, research interested in the sequential patterns that occur within social play has shown that children tend to follow normative play patterns during group interactions and that these patterns are stable across gender and socioeconomic status^41^. While it is thus apparent that children are acquiring the skills necessary to recognize and apply patterned information to their social world which are crucial to engage in appropriate social interactions, the role that statistical learning plays in this process, and how it relates to competency in social interactions, has not been directly tested. It is therefore important to investigate the potential relationship between statistical learning and social competency to mitigate the amount of social-infringing behaviours that may be a result of an impaired ability to learn patterns and probabilities that guide successful social interactions.

Studies have began exploring the converse of this relationship - that a decreased ability to learn patterns or relationships that would otherwise enhance language learning could result in social competency deficits. One such empirical testbed for this idea is Autism Spectrum Disorder (ASD). ASD is a neurodevelopmental disorder associated with social communication difficulties^42^. Social communication issues in autism can include difficulties with turn taking during conversation, maintaining eye contact, and understanding the use of non-verbal language. Although not explicitly part of the diagnostic criteria, research has consistently shown that autistic individuals also show impairments related to both the comprehension and production of language^40,43–46^. To date, studies that have explored auditory and visual statistical learning in ASD have reported mixed findings with some studies showing impaired learning^8,9,47^ and others showing intact learning relative to age-matched controls^48–51^. In the visual domain, research has demonstrated that after a learning phase, autistic individuals showed atypical electrophysiological markers of visual statistical learning relative to non-ASD controls^8^, and that these markers in the autistic participants significantly correlated with their levels of adaptive social functioning. Importantly, this research shows that visual statistical learning is important for social functioning in autistic individuals when learning is measured at the neural level. Conversely, other research examining visual statistical learning has found that adults with autism demonstrated enhanced visual statistical learning abilities when compared to age-matched controls^49^. In the auditory domain, one study did not find differential behavioural statistical learning abilities in autistic individuals relative to controls and additionally failed to find an association between statistical learning and language in the autism group^51^. With that said, it should be noted that the study was relatively underpowered for a correlational analysis (*n* = 17, largest observed power = 0.19). In contrast, another study exploring statistical learning in the auditory domain using functional magnetic resonance imaging (fMRI) found that autistic individuals show decreased responses in the basal ganglia and left supramarginal gyrus during a sequential statistical learning task when compared to non-ASD controls, decreases which correlated with the degree of communication deficits in those with ASD (Scott-Van Zeeland *et al*., 2010).

Autistic individuals also have been described as having a more ‘detail-focused’ cognitive style where they tend to adopt a domain-general processing style by focusing on the fine details or specific parts of a situation (here being individual syllables) rather than the ‘bigger picture’ (here being tri-syllabic pseudowords)^52,53^. This processing style was initially described in the Weak Central Coherence theory^52,54,55^, and subsequently the Enhanced Perceptual Functioning theory of autism^56–58^. The idea of local processing as a default cognitive style in autism suggests that when left uninstructed, individuals with autism tend to default towards focusing on details or notice features about objects or situations that others may find insignificant. Therefore, tasks that require individuals with autism to ignore meaning or the ‘bigger picture’ and focus on details or finer parts will result in superior performance^59^. Conversely, tasks that require these individuals to extract smaller pieces of information and process that as a whole, meaningful unit will result in impaired performance^60^. It should be noted that recent conceptualizations of this locally-focused cognitive style also suggests that individuals with autism can in fact process at the global level when instructed to do so^56,58^. Given that participants in the current study were not specifically instructed to attend to the Gestalt “words”, we are unable to differentiate between these two theories. These clinical findings and theories provide some converging evidence towards the hypothesis that statistical learning abilities may be impaired in autistic individuals and relate to their social skills, but to date have been equivocal.

Despite past research demonstrating a clear link between statistical learning and language, no studies to date have attempted to understand the contribution of both auditory and visual statistical learning on language and social competency skills. This is surprising given that auditory and visual processing are among the most commonly studied modalities in both autism and statistical learning research. Further, while it is predicted that statistical learning would be related to both language and social competencies, it is an open question as to the directionality of this relationship. That is, it is unclear whether statistical learning directly impacts both language and social competency or if statistical learning impacts language abilities, which then in turn affect social competencies.

We aim to address these open questions in the field with our current study. First, we will explore whether a decreased ability to learn auditory and visual statistics are related to receptive language abilities by administering well-established auditory and visual sequence learning paradigms (henceforth referred to as simply *auditory statistical learning* and *visual statistical learning*, respectively) paired with a clinically valid measure of receptive language. Our participants will include non-ASD undergraduate students between 16 to 21 years of age. Our *a priori* prediction is that auditory and visual statistical learning abilities would be directly related to receptive language abilities. Next, we will examine whether statistical learning abilities are related to social competency skills by administering a clinically-validated measure of social competency with the *a priori* prediction that statistical learning abilities would also be directly related to social competency. Importantly, we will examine the directional three-way relationship between statistical learning, language, and social abilities, if applicable. We are interested in how the ability to learn patterns overall is related to language and social competency skills rather than the domain in which the learning occurs. It is therefore expected that auditory and visual statistical learning will contribute similarly to participants’ language and social competency skills given that both tasks assess the ability to learn statistical patterns in general. Further, our predictions stem from statistical learning in both auditory and visual domains outward to areas related to language and social functioning. Our expectation then, is that impaired statistical learning will have cascading effects on language and social skills.

Finally, given the previously found impairments in all three of these abilities in ASD, we will assess whether statistical learning abilities in the auditory and visual domains are related to autistic symptomatology, with the *a priori* prediction that individuals with higher levels of autistic symptomatology will show lower levels of statistical learning. We will also explore possible three-way relationships between statistical learning, autistic traits, and language if applicable based on our findings. To achieve good statistical power for detecting relationships between these variables, we took advantage of the fact that, as a spectrum disorder, traits associated with ASD are not confined to clinical populations but are observed at non-clinical levels in the general population. Indeed, several measures have been used to examine the way autistic traits present in the general population, including the Autism Quotient AQ^61^; and the Broad Autism Phenotype Questionnaire BAPQ^62^, which have shown to generalize experimental findings in non-ASD to similar findings in ASD populations^59,63–65^. The inclusion of both the AQ and BAPQ to measure autistic traits was based on the notion that first, it is important to use multiple informants when assessing autistic traits, and second, each instrument provides important information that allows us to examine a richer set of autistic traits in a non-ASD population.

## Method

### Participants

Prior to exclusion, the initial sample consisted of 104 adults recruited from the undergraduate psychology pool at the University of Western Ontario as part of a larger study^66^ who received course credit for study completion. Here, we use the research domain criteria (RDoC) framework with the aim of assessing the entire spectrum of levels of ASD that range from clinical disorders to issues at sub-clinical levels. The RDoC framework allows researchers to incorporate different ways of assessing mental disorders through use of genetics, cognitive science, and neuroimaging techniques^67,68^. Participants were therefore recruited using the RDoC framework and were not required to have a formal ASD diagnosis. The final sample consisted of 95 undergraduate students between 16 to 21 years of age (*Mean age* = 18.18, *SD* = 0.73), 61 (64.2%) participants being female (see *Analysis* for exclusionary criteria). All participants were English speakers who had normal or corrected-to-normal hearing and vision. Ethics approval for all study procedures and materials was obtained by the University of Western Ontario Non-Medical Research Ethics Board. All methods were performed in accordance with the relevant guidelines and regulations and written informed consent was obtained from all study participants.

### Materials and procedure

Participants were tested individually in a quiet computer testing room seated approximately 60 cm away from a computer monitor. Participants first completed well-established auditory and visual statistical learning tasks, followed by a clinical language assessment, a questionnaire measure of social competency, and autism symptomatology questionnaires. For both statistical learning tasks, participants verbally received the instructions for the task from a trained researcher.

#### Auditory statistical language learning paradigm

The statistical language learning task is based on that from Saffran *et al*. (1997) in order to make current findings comparable to previously conducted studies^15,66^. Participants first completed a language exposure phase. In the language exposure phase, participants were exposed to a structured, unsegmented language stream for 21 minutes and instructed to colour with a colouring book and colouring pencils until the sounds stopped playing. The language consisted of six tri-syllabic nonsense “words”: *tutibu, babupu, bupada, pidadi, patubi, and dutaba*. There were no acoustic markers to indicate word boundaries between words. However, within the language stream, there were higher transitional probabilities within words (1.0 or 0.33) than between words (0.1 or 0.2).

The language stimuli were constructed from audio recordings of a female native-English speaker using a neutral vocal affect. Recordings were made in a double-walled sound booth with a pedestal microphone (AKG C 4000B) located approximately 30 cm from the speaker’s mouth and routed to a USBPre 2 pre-amplifier (Sound Devices) using SpectraPlus software (Pioneer Hill Software, 2008). Recordings were made of each of the 12 target syllables in the middle of a three-syllable sequence, within every coarticulation context required for the language. Eight repetitions of each sequence were recorded, and the token with the most neutral pitch contour and best sound quality was chosen and uploaded into Sound Forge Audio Studio (Sony Creative Software, *version* 10.0) editing software. Middle syllables from the recorded tokens were extracted by identifying the final offset of vowel oscillation in the previous syllable to the offset of vowel oscillation in the target syllable. These were then concatenated to create the final 21-minute stream of words. The stream consisted of 360 tokens of each word in random order, with no word presented twice in sequence. The language maintained a consistent speech rate (average 5.1 syllables/s) using a time stretch and was normalized to a pitch of F0 = 196 Hz using the pitch shift in Sound Forge Audio Studio. There were no pauses between words; as such, the only cues to word boundaries were the lower transitional probabilities for between-word syllable pairs. Language stimuli were presented using E-Prime 2.0.10^69^ through speakers or headphones at a comfortable listening volume.

Immediately following the artificial language exposure, participants completed a two-alternative forced-choice test (2AFC) to assess whether they could identify trained words from the artificial language. For each test item, participants heard a trained word from the artificial language paired with a nonword foil, separated by 500 ms of silence. Nonword foils were constructed from the same syllable inventory as the words from the artificial language, with the constrained that the transitional probabilities between syllables were 0.0: *pubati, tapudi, dupitu, tipabu, bidata, batipi*. The foil words were recorded in the same manner as the trained words. Each of the six trained words was paired exhaustively with each of the six foil words, comprising 36 total test items. For the 2AFC test, instructions were displayed on the computer screen, asking participants *‘which of these two words sounds most like something you heard in the language’*. Participants indicated their response on a keyboard by pressing the “*A*” or “*L*” key for the first or second “word”, respectively. Test instructions remained on the screen throughout the test phase. Correct responses were counterbalanced and equally presented in the first or second presentation to avoid response bias. Overall accuracy on the 2AFC were analyzed.

#### Visual statistical learning paradigm

**Figure 1:**
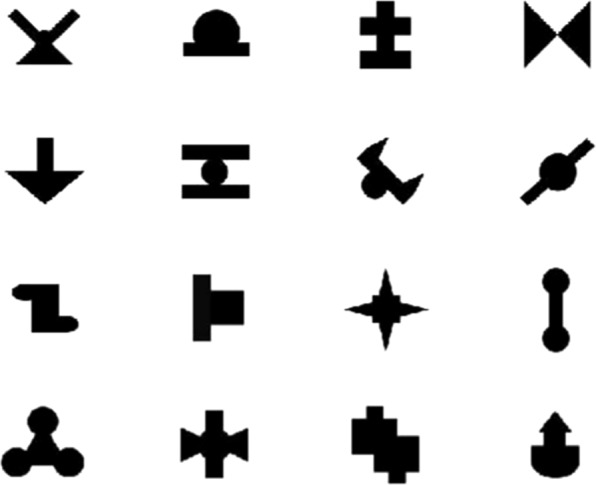
16 novel shapes used in visual paradigm.

**Figure 2:**
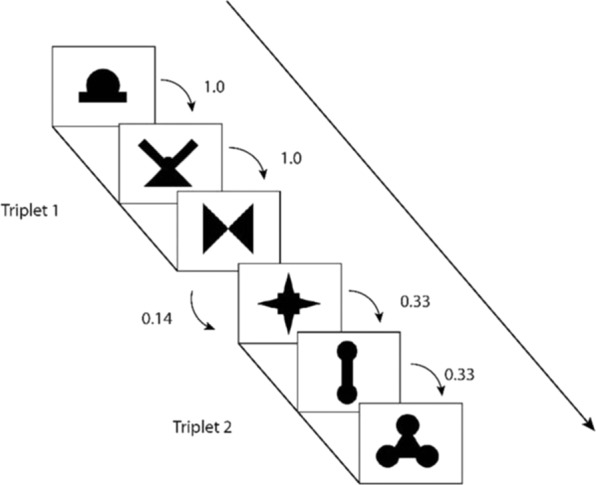
Example of probabilities within and between triplets.

Next, participants completed the visual statistical learning task, which was identical to the established paradigm used by Siegelman, Bogaerts and Frost (2016) in order to make current findings comparable to previously conducted studies^22,66^. During this task, participants were seated in front of a computer screen, while novel shapes were presented one at a time (Fig. 1). As with the artificial language stimuli, the shapes were presented in a structured, unsegmented sequence. During the learning phase, participants were instructed to watch the stream of shapes and try their best to remember them. The shapes were organized into eight triplets that differed according to their within-triplet transitional probabilities, with four triplet items having transitional probabilities of 0.33 and the other four having transitional probabilities of 1.0. Between-triplet transitional probabilities were 0.14 or less (Fig. 2). Participants passively viewed the stream of shapes for 10 minutes, with each triplet sequence appearing 24 times. Shapes were presented for 800 ms, with a 200 ms inter-stimulus interval (ISI).

The test phase began immediately following the learning phase. The test phase used in the current study was identical to that used by Siegelman *et al*. (2016), and consisted of 42 test items of varying difficulty, presented one at a time. This task differed from previous statistical learning tasks in that test items required different responses as well as differed from one another in terms of item difficulty and the number of response options. The first block of 34 trials were pattern recognition items, and required participants to pick the familiar, or trained, sequence from either two (2AFC) or four (4AFC) possible responses. Pattern recognition items included both pairs of shapes (part triplets) and triplets. For the 24-items that contained triplet sequences, participants were instructed to *‘choose the pattern that appeared together in the first part’*, sixteen being 2AFC and eight 4AFC. For the ten items that contained pairs of shapes participants were instructed to *‘choose the pattern that you are most familiar with as a whole’*, six being 2AFC and four 4AFC. Instructions on recognition items therefore differed depending on whether the participant was presented with triplet or part triplet sequences. All responses were numbered, and participants indicated their response by clicking the corresponding key on a keyboard. The next eight trials were pattern completion items. For these trials, participants were shown an incomplete trained sequence, and selected the shape that best completed the sequence from a set of three possible responses. Participants were instructed to *‘choose the shape that best completes the pattern’* from the 3AFC items, which included four triplet-completion items and four pair-completion (that is, part-triplet) items.

For all test trials, there was only one triplet or part-triplet that followed the statistical regularities presented in the test phase. Accuracy scores were collected and analyzed for all items.

#### Standardized language measure

Participants receptive and expressive language abilities were examined by administering six subtests from the *Clinical Evaluation of Language Fundamentals* CELF-5^70^, with our focus being on receptive language. The CELF-5 is a commonly used language measure that is sensitive to receptive and expressive language abilities of child and adult clinical populations including specific language impairments^71^, attention deficit hyperactivity disorder^72^, and ASD^73^. Three subtests were administered to examine receptive language abilities and included *Word Classes, Understanding Spoken Paragraphs*, and *Semantic Relationships* and three subtests examined expressive language abilities, and included *Formulating Sentences, Recalling Sentences*, and *Sentence Assembly*. Standardized receptive and expressive language index scores were then calculated for each participant by summing the three subtests in each domain, as prescribed in the protocols for the language assessment measure.

#### Assessment of social competency

The Multidimensional Social Competence Scale MSCS^74^; is a seventy-seven-item measure in which participants indicated on a five-point Likert scale (not true/almost never true; rarely true; sometimes true; often true; very true/almost often true) a response to statements on social motivation, inferencing, knowledge, empathy, verbal and non-verbal skills and emotion regulation. In addition to receiving a total social competency score, participants received a score on seven subscales assessing social motivation, social inferencing, empathy, social knowledge, verbal conversational skills, nonverbal sending skills, and emotion regulation. Example items on this scale include, *“I prefer to spend time alone (e.g., I am most content when left on my own)”, “I recognize when people are trying to take advantage of me”, “I change my behaviour to suit the situation. For example, I might be more polite/formal around authority figures like teachers or supervisors but be more casual around peers”*.

#### Measures of autistic traits and symptom severity

Several well-established questionnaires used as screening protocols for ASD symptoms were administered. Questionnaires were completed, and responses were recorded using the Qualtrics Survey Software. The Autism Quotient AQ^61^; is a fifty-item measure using a four-point Likert scale (definitely disagree; slightly disagree; slightly agree; definitely agree) to assess the degree to which an individual has traits associated with autism. The Broad Autism Phenotype Questionnaire BAPQ^62^; is a thirty six-item questionnaire that was completed to measure autistic traits. Participants indicated a response on a six-point Likert scale (very rarely; rarely; occasionally; somewhat often; often, very often) on a variety of personality statements.

### Analysis

Items on the auditory and visual statistical learning tasks were analyzed for accuracy and above-chance performance. As a 2AFC, the chance rate of the test-phase of the auditory statistical learning task was 50%, whereas the visual statistical learning test phase included 2AFC, 3AFC, and 4AFC components, thus having chance levels of 50%, 33%, and 25%, respectively. Overall accuracy scores from the auditory statistical learning task were calculated by averaging participants’ correct responses on all items. Visual statistical learning accuracy included averaging the items on each separate component followed by averaging correct responses on all 42 test items to create an overall mean accuracy score. Overall mean accuracy scores from both tasks as well as mean accuracy scores from the separate components on the visual statistical learning task were then used to examine possible relationships between statistical learning, receptive and expressive language, social competency, and autistic traits.

The CELF-5 language assessment is intended to assess receptive and expressive language abilities from individuals aged 5.0 to 21.11. Therefore, participants above this age were excluded from the analyses (*N* = 5). Those identified as outliers (±2 SD from the mean values; *N* = 3) on total questionnaire measures including the MSCS, AQ, and BAPQ, rather than questionnaire subscale measures were also excluded. One additional participant was excluded for not completing the visual statistical learning task.

The MSCS was used to explore the relationships between social competency, language, and statistical learning. Participants total scores assessing social competency as well as subscale scores measuring social motivation, social inferencing, empathy, social knowledge, verbal conversational skills, nonverbal sending skills, and emotion regulation were analyzed.

Lastly, total scores on the AQ and BAPQ were used to explore the relationship between autistic traits and statistical learning as well as determine the range of scores observed.

All data were normally distributed and therefore, Pearson bivariate correlations were used to explore the possible relationship between statistical leaning and language, statistical learning and social competency, and statistical learning and autistic traits. The Benjamini-Hochberg false discovery rate procedure was used to adjust for multiple comparisons with a false discovery rate of 25%. Finally, mediation analyses were conducted on all social competency scales that were significantly correlated with both receptive language and a measure of statistical learning. Specifically, visual statistical learning acted at the independent variable, receptive language as the mediator, and social knowledge as the dependent variable in the first mediation analysis. The second mediation analysis was identical, with the exception that nonverbal sending skills acted as the dependent variable.

## Results

### Above chance performance on statistical learning tasks

Performance was found to be reliably above chance for overall auditory (64%, *t*_(94)_ = 57.08, *p* < 0.001, *Cohen’s d* = 5.82) and visual (58%, *t*_(94)_ = 42.68, *p* < 0.001, *Cohen’s d* = 4.46) statistical learning tasks. In addition, performance was reliably above chance for visual pair completion (59%, *t*_(94)_ = 23.52, *p* < 0.001, *Cohen’s d* = 2.36), triplet completion (55%, *t*_(94) = _20.55, *p* < 0.001, *Cohen’s d* = 2.12), 2AFC recognition (68%, *t*_(94)_ = 43.61, *p* < 0.001, *Cohen’s d* = 4.53), 4AFC recognition (41%, *t*_(94)_ = 18.38, *p* < 0.001, *Cohen’s d* = 1.86), 2AFC pair recognition (64%, *t*_(94)_ = 28.61, *p* < . 001, *Cohen’s d* = 2.91), and 4AFC pair recognition (44%, *t*_(94)_ = 21.18, *p* < 0.001, *Cohen’s d* = 2.20). See Table 1 for descriptive statistics.

**Table 1:** Descriptive statistics for auditory and visual statistical learning paradigms.

	Mean *(SD)*	Variance (σ)	Minimum	Maximum
1. Auditory Accuracy	0.64 *(0.11)*	0.01	0.39	0.83
2. Visual Accuracy	0.58 *(0.13)*	0.02	0.26	0.92
3. Visual Pair	0.59 *(0.24)*	0.06	0.00	1
4.Visual Triplet	0.55 *(0.26)*	0.07	0.00	1
5. Visual 2AFC Recognition	0.68 *(0.15)*	0.02	0.25	0.94
6. Visual 4AFC Recognition	0.41 *(0.22)*	0.05	0.00	0.88
7. Visual 2AFC Pair Recognition	0.64 *(0.22)*	0.05	0.17	1
8. Visual 4AFC Pair Recognition	0.44 *(0.20)*	0.04	0.00	0.75

### Relating statistical learning and language

Visual statistical learning accuracy was found to be significantly correlated with receptive language ability (*r*_(94)_ = 0.31, *p* = 0.002) indicating that decreased visual statistical learning abilities are related to decreased receptive language abilities. However, contrary to our predictions, auditory statistical learning was not found to be significantly related to receptive language ability (*r*_(94)_ = 0.08, *p* = 0.45).

### Relating statistical learning and social competency

**Figure 3:**
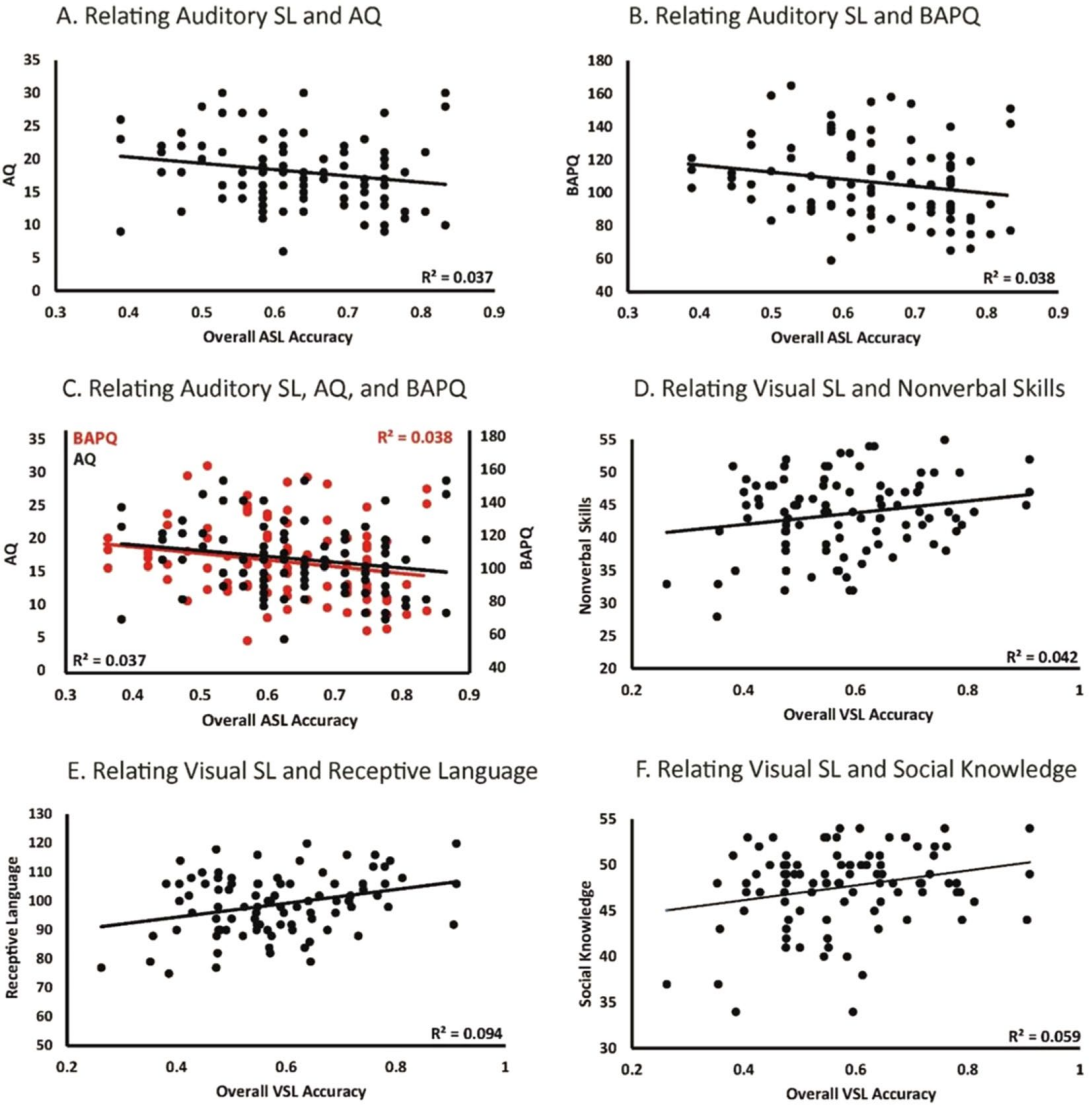
Relating (**A**) auditory statistical learning and total AQ scores (**B**) auditory statistical learning and total BAPQ scores (**C**) auditory statistical learning, total AQ (black), and total BAPQ (red) scores (**D**) visual statistical learning and MSCS nonverbal sending skills (**E**) visual statistical learning and receptive language (**F**) visual statistical learning and MSCS social knowledge.

Visual statistical learning was not significantly related to total social competency scores (*r*_(94)_ = 0.18, *p* = 0.09), nor the MSCS subscales social motivation (*r*_(94)_ = −0.08, *p* = 0.45), social inferencing (*r*_(94)_ = 0.12, *p* = 0.26), verbal conversation skills, (*r*_(94)_ = 0.09, *p* = 0.41), or emotional regulation (*r*_(94)_ = 0.09, *p* = 0.41). However, it was found to be significantly related to social competency skills including empathy (*r*_(94)_ = 0.26, *p* = 0.01), social knowledge (*r*_(94)_ = 0.24, *p* = 0.02), and nonverbal sending skills (*r*_(94)_ = 0.20, *p* = 0.05) in that increased visual statistical learning abilities were related to increased social competency skills. However, auditory statistical learning was not found to be significantly related to social competency. Please see Table 2 for correlation values for all variables as well as Fig. 3 for a visualization of these relationships.

**Table 2:** Bivariate correlations between Statistical Learning, Language, Social Competency, and Autistic Traits (N = 95).

	1	2	3	4	5	6	7	8	9	10	11	12	13	14
1. Auditory Accuracy	—	0.267	0.447	0.334	0.217	0.246	0.258	0.149	0.861	0.462	0.284	0.485	0.004**	0.001**
2. Visual Accuracy	0.12	—	0.002**	0.181	0.088	0.012*	0.018*	0.047*	0.452	0.264	0.405	0.414	0.627	0.775
3. Receptive	0.08	0.31**	—	0.000**	0.014*	0.392	0.001**	0.013*	0.431	0.018*	0.013*	0.044*	0.863	0.192
4. Expressive	0.10	0.14	0.55**	—	0.054	0.025*	0.001**	0.038*	0.357	0.664	0.047*	0.394	0.070	0.769
5. MSCS Total	0.13	0.18	0.25*	0.20	—	0.000**	0.000**	0.000**	0.000**	0.000**	0.000**	0.000**	0.003**	0.001**
6. *Empathy*	0.12	0.26*	0.09	0.23*	0.65**	—	0.000**	0.000**	0.000**	0.000**	0.011*	0.439	0.795	0.185
7. *Social Knowledge*	0.12	0.24*	0.33**	0.34**	0.71**	0.51**	—	0.000**	0.311	0.000**	0.000**	0.000**	0.370	0.297
8. *Nonverbal Sending Skills*	0.15	0.20*	0.26*	0.21*	0.66**	0.56**	0.46**	—	0.000**	0.001**	0.036*	0.230	0.081	0.086
9. *Social Motivation*	−0.02	−0.08	−0.08	−0.10	0.59**	0.42**	0.11	0.49**	—	0.000**	0.446	0.125	0.002*	0.000**
10. *Social Inferencing*	0.08	0.12	0.18	0.05	0.77**	0.41**	0.49**	0.34*	0.40**	—	0.000**	0.000**	0.007*	0.002*
11. *Verbal Skills*	0.11	0.09	0.25*	0.21*	0.68**	0.26*	0.56**	0.22*	0.08	0.46**	—	0.000**	0.092	0.048
12. *Emotion Regulation*	0.07	0.09	0.21*	0.09	0.65**	0.08	0.40**	0.12	0.16	0.54**	0.59**	—	0.007*	0.043
13. AQ	−0.29**	0.05	0.02	−0.19	−0.30**	−0.03	−0.09	−0.18	−0.31**	−0.27**	−0.17	−0.28**	—	0.000**
14. BAPQ	−0.32**	−0.03	0.14	−0.03	−0.34**	−0.14	−0.11	−0.18	−0.37**	−0.32**	−0.20*	−0.21*	0.67**	—
*Mean*	0.64	0.58	98.75	101.59	294.43	43.99	47.62	43.64	40.47	41.57	39.10	38.04	18.01	106.31
*S.D*.	0.11	0.13	10.36	8.35	27.64	5.18	4.39	5.87	6.83	5.54	6.31	7.21	5.40	24.28

Note. *p < 0.05; **p < 0.01. Correlation values are presented on the bottom left and corresponding p values are presented on the upper right. P-values listed as 0.000 represent p-values < 0.001. Mean and SD are variable descriptives.

### Receptive language as a mediating variable

**Figure 4:**
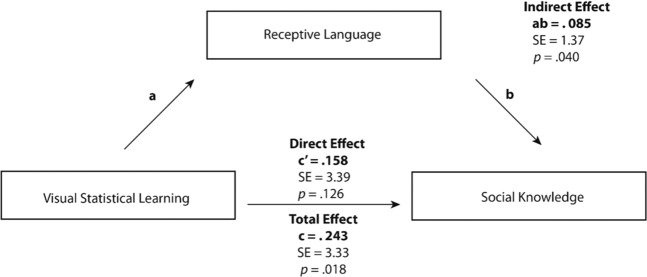
Mediation model testing the effect of receptive language ability on visual statistical learning and social knowledge.

Both visual statistical learning and receptive language were significantly correlated with the social competency subscale of *Social Knowledge*. As such, a series of multiple linear regression analyses were used to determine if receptive language ability significantly mediated the relationship between visual statistical learning abilities and social knowledge (Fig. 4). Prior to completing these analyses, the following conditions were shown to be true: (1) statistical learning was significantly related to social knowledge, (2) statistical learning was significantly related to receptive language, (3) receptive language was significantly related to social knowledge after controlling for statistical learning, and (4) the impact of statistical learning on social knowledge was significantly less after controlling for receptive language abilities. Our regression analyses showed that receptive language had full mediating effects on social knowledge. The relationship between visual statistical learning and social knowledge not only became statistically non-significant, but also exhibited a significant drop in predictive nature when receptive language ability was added into the model. The indirect effect (ab = 0.085, *SE* = 1.37, 95% [0.945, 6.143]) was judged to be statistically significant using the Sobel (1983) test (*z* = 2.05, *p* = 0.040). Thus, once the contribution of receptive language ability was accounted for, visual statistical learning only significantly contributed to social knowledge indirectly, through receptive language. Additionally, receptive language ability accounted for 7% (R^2^ = 0.069) of participants social knowledge scores and visual statistical learning abilities accounted for 2% (R^2^ = 0.023) of participants social knowledge scores. Combined, receptive language and visual statistical learning account for 13% (R^2^ = 0.128) of the variance in our samples social knowledge scores (*F*_(2, 92)_ = 6.77, *p* = 0.002).

To test the directionality of this relationship, an additional mediation analysis was performed to determine whether receptive language specifically mediated the relationship between visual statistical learning and social knowledge, or if social knowledge could also have acted as a mediator between the two variables. The mediation analysis revealed that when social knowledge was included as a mediator between visual statistical learning and receptive language, the indirect pathway (ab = 0.065, *S*E = 2.80, 95% [1.181, 12.029]) was only marginally significant (*z* = 1.80, *p* = 0.072), therefore providing evidence of directionality as proposed in the original model. It should be noted, however, that the total effect predicting receptive language abilities from visual statistical learning was statistically significant, further confirming that the overall effect of visual statistical learning on receptive language was statistically significant. Our data therefore suggest that there is a directional relationship between visual statistical learning abilities, receptive language, and social knowledge where receptive language specifically mediates this relationship.

**Figure 5:**
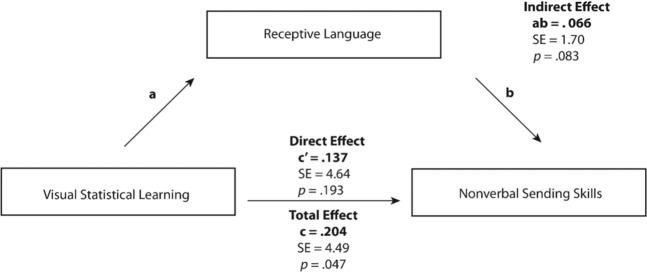
Mediation model testing the effect of receptive language ability on visual statistical learning and nonverbal sending skills.

Additionally, the social competency subscale of *Nonverbal Sending Skills* was significantly related to both receptive language and visual statistical learning. The mediation was performed in an identical manner to the first where the following conditions were shown to be true: (1) statistical learning was significantly related to social knowledge, (2) statistical learning was significantly related to receptive language, (3) receptive language was significantly related to nonverbal sending skills after controlling for statistical learning, and (4) the impact of statistical learning on nonverbal sending skills was significantly less after controlling for receptive language abilities. As such, we conducted a regression to test whether receptive language mediated the relationship between visual statistical learning and nonverbal sending skills (Fig. 5), which revealed a partially mediated pathway (ab = 0.066, *SE* = 1.70, 95% [0.470, 6.692], *z* = 1.73, *p* = 0.083) where the direct effect (the remaining effect of visual statistical learning on nonverbal sending skills when receptive language has been included in the analysis) was not significant once receptive language was included. Thus, when the contribution of receptive language was accounted for, visual statistical learning only contributed significantly to nonverbal sending skills indirectly, through receptive language. Similar to the above mediation model, the total effect predicting nonverbal sending skills from visual statistical learning was statistically significant, indicating that visual statistical learning abilities do significantly predict receptive language abilities.

### Range of autistic traits

Our results showed that total sores on the AQ ranged 6 to 30 and average responses on the individual items on the BAPQ ranged from 1.64 to 5.33 where a cut-off value of 32+ on the AQ and an average of 3.14 across all 36 individual items on the BAPQ is useful in distinguishing individuals with clinical levels of autistic traits^61,62^.

### Relating statistical learning and autistic traits

Auditory statistical learning was found to be significantly related to total scores on the AQ (*r*_(94)_ −0.29, *p* = 0.004) and BAPQ (*r*_(94)_ = −0.32, *p* = 0.001) suggesting that, as predicted, decreased statistical learning abilities in the auditory domain are related to increased autism symptom severity. However, this same relationship was not observed for overall visual statistical learning (AQ: *r*_(94)_ = 0.05, *p* = 0.63, BAPQ; *r*_(94)_ = −0.03, *p* = 0.78), or any of the additional visual statistical learning scores examining pattern recognition and completion. Given that mediation analyses can only be carried out on three variables that are related, no further mediation analyses were conducted involving autistic traits.

## Discussion

The purpose of this study was to examine the relationship between statistical learning, language, social competency, and autistic traits, and a number of novel relationships were observed. First, our data partially confirmed our *a priori* hypothesis that visual statistical learning abilities are directly related to both increased language comprehension and social competency abilities. Second, our mediation results provide support for a specific directional relationship between visual statistical learning abilities, receptive language, and social competency such that the relationship between visual statistical learning and social competency is mediated by receptive language abilities. Third, while auditory and visual statistical learning were both significantly above chance levels, these two measures of statistical learning were independent of each other, suggesting that separate processes underlie each. Finally, our data support the hypothesis that statistical learning in the auditory domain, but not the visual, is negatively correlated with autistic traits such that individuals with higher levels of autistic traits exhibited weaker auditory statistical learning.

Consistent with previous research, our results show that visual statistical learning abilities are related to receptive language^75^ in that an increased ability to learn patterns in the visual domain is related to increased language comprehension. It was also found that increased visual statistical learning abilities were related to increased social competency skills, suggesting that visual statistical learning is important for understanding the emotions of oneself and others, how to act appropriately in social situations, as well as using appropriate nonverbal communication. Indeed, it has been suggested that successful social interactions depend on a strong ability to pick up on temporal patterns that occur in facial expressions, gestures, and other non-verbal cues^76^. This ability to pick up on subtle sequences of non-verbal information allow us to draw inferences about others emotional states and intentions during social interactions. Previous research has linked visual statistical learning and social abilities including adaptive social functioning^8^. However, this relationship, to our knowledge, has only been reported in ASD populations. This is therefore the first study to identify a relationship between visual statistical learning, language, and social abilities in non-ASD individuals.

One novel finding that has not yet been reported in the literature was that the relation between visual statistical learning and social knowledge was significantly mediated by receptive language ability. Rather than a direct relationship between visual statistical learning abilities and social knowledge, language comprehension therefore helps to clarify the observed relationship between these two variables. Our mediation results therefore show that visual statistical learning abilities impact receptive language abilities which in turn, impact social knowledge. This same directional relationship was not observed when social knowledge was included as the mediator rather than the dependent variable, supporting the directional relationship between visual statistical learning, receptive language, and social knowledge. Further, when examining whether receptive language significantly mediates the relationship between visual statistical learning and nonverbal sending skills, the mediated effect was found to be marginally significant, indicating that some of the variability in our model is explained by this indirect pathway and further investigation into the nature of this mediated relationship is warranted. Although prior research has linked statistical learning and language in non-ASD individuals, only those using ASD populations have previously found correlational links between auditory^9^ and visual^8^ statistical learning and social competency skills. Our results therefore provide novel evidence, showing that this same relationship between statistical learning and social competency is apparent in non-ASD populations. Taken together, these findings highlight the importance of language in understanding the relationship between statistical learning and social competency in the general population. Also, our findings highlight the importance of examining both total and subscale scores of social competencies to understand the distinct relationships they can have with statistical learning and language comprehension.

It is surprising that we did not find a relationship between auditory statistical learning and language abilities, as auditory statistical learning has been shown to be associated with vocabulary^26^ and other broader language skills^25^. There are a number of possible reasons for this discrepant result. Perhaps the clearest distinction between this work and previous research is that most research that has linked auditory statistical learning and language abilities has focused on infants or young children^26,33,37–39,77^, while in the current study, data was from adult participants with fully-developed language abilities. It may be that statistical learning is an important predictor of early language development or in the rate of language development, but that this relationship diminishes as language development plateaus. Likewise, infant studies use differing metrics of statistical learning, such as looking time measurements, while in the current study, learning was measured by recording explicit responses from adult participants. The discrepancy between our findings and those of previous studies relating auditory statistical learning and language could therefore be attributed to subtle task differences in addition to using different measures of learning.

An alternative explanation is that attention is driving the relationship between visual statistical learning and receptive language abilities. It has been found that attention influences the relationship between visual statistical learning abilities and receptive language at both the behavioural and neurophysiological level^75^. Moreover, auditory statistical learning may depend, at least in part, on attention^78^, and is similarly involved in visual statistical learning^79^. Attention helps language users map words, sentences, and images onto their appropriate referents and before they can accomplish this, they must first attend to the patterns that are occurring in their environment^80^. Here, our methodology mirrored previous paradigms in both the auditory and visual domains. The previously established visual paradigm includes instructing participants to *“try their best to remember the shapes that were presented”*^22^ while participants actively attended the presentations, whereas in the auditory paradigm, participants were simply instructed to *“colour until the sounds stopped playing”* and were thus actively attending to a secondary task as opposed to the auditory stimuli^15^. Thus, attention may have impacted subsequent learning from our visual statistical learning task, as well as the relationship between our visual statistical learning measure and receptive language abilities.

Likewise, we found that only visual statistical learning was significantly related to social competency, in contrast to our hypothesis that learning patterns in general should be important for predicting the actions of others and relying on previous patterns and relationships during social interactions to help guide current ones. While the aforementioned impact of attention is a possible explanation here there are other possibilities that should be considered. For instance, it could be that given that the visual and auditory statistical learning tasks differed in their probabilities, and the way learning was measured, the visual statistical learning task used here may be more closely related to the complexities of real social interactions. As a result, sequences in the visual task could be less predictable than those presented in the auditory task.

Auditory and visual statistical learning were also shown to be independent of one another which aligns well with previous research^81^ s do exist and are not uniform across auditory and visual modalities or across stimuli. Therefore, it could be that statistical learning is dependent on the sensory modality of the input. For e. Individual sensitivities to the probabilities that occur in auditory and visual task xample, auditory statistical learning abilities may be more important for aspects of language outside of those measured here, where visual abilities may be important for non-verbal abilities, such as learning the unspoken rules of social language. The results from our study provide support for this difference and suggest that distinct processes may underlie how auditory and visual patterns are processed. It is however, important to note again that our statistical learning tasks differed from one another, making it difficult to determine whether the lack of relationship between the two tasks is a result of methodological differences or the modality in which learning occurred. The complexity of the responses to evaluate learning in the two tasks differed considerably and as a result, it is difficult to say whether these methodological differences are interfering with our interpretation of these relationships.

In addition to auditory and visual statistical learning differing in their relationship with receptive language and social competencies, and not relating to one another, they were also differently related to ASD traits. Those with higher autistic traits tended to perform worse on the auditory statistical learning task, which is consistent with other studies investigating statistical learning in ASD using similar auditory statistical learning tasks that require implicit, sequence learning^8,9^. Visual statistical learning, however, was not shown to be related to autistic traits despite being related to autism-related social competency. Given the apparent social competency difficulties in autism, it is surprising that we found a relation between visual statistical learning, language, and social competency but not visual statistical learning and autistic traits. Research examining visual processing in autism has reported enhanced performance on visual tasks that require both detection and discrimination however, this enhanced performance is limited to simple over complex visual stimuli^82,83^. Comparable visual processing abilities in non-ASD and ASD individuals could therefore explain why auditory statistical learning was more strongly related to autistic traits than visual statistical learning abilities.

Further, the idea of Enhanced Perceptual Functioning as a default cognitive style in autism^54,56–58^ could also explain why we saw different relationships between autistic traits and our auditory and visual statistical learning tasks. As mentioned above, this theory posits that when individuals with autism are not instructed, they tend to focus on finer details of an object or situation however, when these same individuals must extract smaller pieces of information and process this information as a whole, they tend to show impaired performance. In our auditory statistical learning task, we did not instruct our participants to pay attention to the patterns. Instead, we had them passively listen to the sounds while engaged in a colouring task. As a result, participants may have focused more on the finer details during this task instead of processing the patterns which could explain our finding that autistic traits were related to auditory statistical learning abilities. However, the idea of Enhanced Perceptual Functioning as a cognitive style also suggests that individuals with autism can in fact process at the global level when instructed to do so. Thus, in our visual statistical learning task, where participants were instructed to watch the shapes, they were more likely to focus on the patterns that were presented rather than the individual shapes. Likewise, these instructions could explain why we did not find a relationship between autistic traits and visual statistical learning abilities.

Despite this, during statistical learning, individuals are learning how things are grouped in the environment and there are a number of neurobiological accounts of autism that, in their own different ways, predict an issue with statistical learning. The predictive coding framework suggests that our experiences are influenced by incoming sensory information as well as prior perceptual experiences^53,84^. Atypical sensory perception in autism can be explained through this framework, suggesting that these individuals do not integrate incoming sensory information and prior knowledge in a typical manner, either due to a decreased ability to form reliable probability maps or an overweighting of incoming sensory information. In either case, this framework predicts a decreased ability to utilize patterns and temporal statistics within in the environment. The above-mentioned neurobiological abnormalities that impact cognitive functioning in ASD therefore suggest that autistic individuals are processing and responding to statistical probabilities in the environment differently than their non-ASD peers. However, additional research is needed in order to determine how these individuals are processing these patterns and whether this differential processing could explain the language and social competency difficulties observed in ASD.

### Future directions

Although both auditory and visual statistical learning tasks used in the current study have been well established, there are several areas for improvements that should be considered in future research. First, both statistical learning tasks assess recognition after familiarization, which may reflect more explicit types of learning rather than implicit. Second, the test items in the auditory task are all identical in levels of difficulty and share similar properties, where participants are required to compare two words and pick the one that is most familiar. Addressing these shortcomings will allow researchers to make stronger claims about the implicit nature of statistical learning as well as examine varying levels of abilities that align more closely with real world learning.

Recent work in statistical learning has attempted to improve some of these shortcomings by measuring learning more implicitly, while participants are exposed to the familiarization phase^24,85,86^. In these statistical learning tasks, participants are required to respond to a selected target occurring in a continuous stream of syllables, with faster reaction times indexing better processing of predictable targets. In turn, attention can be monitored by examining accuracy scores (i.e. percentage of correct/incorrect responses can determine guessing). New methodologies such as using ‘temporal community structures’^87,88^ are another potential avenue for future statistical learning tasks. During this task, participants view a continuous stream of stimuli that follows a repeating pattern. Stimuli are randomly assigned to one of three communities where those within the same community co-occur together more often and those that connect one community to another act as boundaries. Thus, the probabilities within communities share the same temporal associations, providing learners with a basis for segmenting the sequences. During the presentation, they are instructed to press a button each time they believe there is a natural break in the pattern. Learning is therefore measured online, while it is occurring. This design therefore allows researchers to measure learning while it is occurring as well as control transitional probabilities in a more consistent manner. These techniques may (1) allow for more direct comparison across sensory modalities, (2) measure statistical learning not only after familiarization, but also the rate of statistical learning during familiarization, and (3) improve the ecological validity of statistical learning tasks by mimicking real world learning conditions that involve more complex temporal relationships than those presented by transitional probabilities used in previous statistical learning tasks.

Given that past research has found evidence for reduced statistical learning abilities in ASD at the behavioral level and associations between statistical learning and neural differences in ASD compared to TD individuals, it might also be beneficial to combine these methods to better understand how statistical learning is processed in autism and other developmental disabilities. Further, using EEG and fMRI methods to examine statistical learning will enable researchers to understand these processes in minimally verbal, low-functioning children and adults, revealing valuable information about pattern learning beyond those provided by behavioral measures alone.

## Conclusions

Here, we have highlighted the important role that statistical learning plays in language ability and social competency. Our results demonstrate a clear relationship between statistical learning, language, and social competency abilities as well as statistical learning and autistic traits. Furthermore, that the relationship between statistical learning and social competency, specifically social knowledge, is mediated by receptive language ability suggesting that impaired statistical learning abilities cascade into language and social competency abilities. Interestingly, this same directional effect was not observed when social knowledge was included as the mediator between visual statistical learning and receptive language. It is important to note that the methodological differences between the auditory and visual statistical learning tasks may have accounted for the differential relationships that emerged between auditory and visual statistical learning and language, social communication, and autism symptomatology. Future work should therefore match statistical learning tasks in order to make conclusions about whether one type of statistical learning is more important for language and social communication and another is more related to autism symptom severity. Further, it is important to note that the relationships observed here are relatively small in magnitude and should therefore be interpreted with caution. Despite this, the results from the current study provide valuable insight into how statistical learning relates to both language and social abilities. Language and social difficulties that are a result of impaired statistical learning can have a negative impact on many aspects of an individual’s life. If untreated, issues related to poor language comprehension and social competency can extend beyond adolescence and into adulthood, causing further problems related to employment and education. With that in mind, research has shown that training programs aimed at improving temporal and sequential processing capacities can bolster the recognition of speech and non-speech temporal sequences in children with language impairments^89^ as well as language processing in non-ASD adults^90^. Therefore, incorporating activities into therapies and interventions that encourage pattern and sequence learning may help non-ASD and ASD individuals learn these patterns and probabilities more readily. In turn, setbacks that result from poor language and social competency can be mitigated^12,91^.
